# GelMA, Click-Chemistry Gelatin and Bioprinted Polyethylene Glycol-Based Hydrogels as 3D Ex Vivo Drug Testing Platforms for Patient-Derived Breast Cancer Organoids

**DOI:** 10.3390/pharmaceutics15010261

**Published:** 2023-01-12

**Authors:** Nathalie Bock, Farzaneh Forouz, Luke Hipwood, Julien Clegg, Penny Jeffery, Madeline Gough, Tirsa van Wyngaard, Christopher Pyke, Mark N. Adams, Laura J. Bray, Laura Croft, Erik W. Thompson, Thomas Kryza, Christoph Meinert

**Affiliations:** 1 School of Biomedical Sciences, Faculty of Health, Translational Research Institute (TRI), Queensland University of Technology (QUT), Brisbane, QLD 4000, Australia; 2 Max Planck Queensland Centre, Brisbane, QLD 4059, Australia; 3 Centre for Biomedical Technologies, QUT, Brisbane, QLD 4059, Australia; 4 Australian Prostate Cancer Research Centre (APCRC-Q), QUT, Brisbane, QLD 4102, Australia; 5 Gelomics Pty Ltd., Brisbane, QLD 4059, Australia; 6 Centre for Personalised Analysis of Cancers (CPAC), Brisbane, QLD 4102, Australia; 7 ARC Training Centre for Cell and Tissue Engineering Technologies, QUT, Brisbane, QLD 4059, Australia; 8 School of Mechanical, Medical and Process Engineering, QUT, Brisbane, QLD 4000, Australia; 9 Mater Research Institute, Brisbane, QLD 4102, Australia; 10 Breast and Endocrine Surgery, Princess Alexandra Hospital, Woolloongabba, QLD 4102, Australia; 11 Centre for Genomics and Personalised Health, Brisbane, QLD 4000, Australia

**Keywords:** 3D cancer model, patient-derived organoid, hydrogel, GelMA, gelatin, polyethylene glycol, extracellular matrix, tumor microenvironment, doxorubicin, adriamycin, paclitaxel, EP31670, breast cancer, drug resistance, precision medicine

## Abstract

3D organoid model technologies have led to the development of innovative tools for cancer precision medicine. Yet, the gold standard culture system (Matrigel^®^) lacks the ability for extensive biophysical manipulation needed to model various cancer microenvironments and has inherent batch-to-batch variability. Tunable hydrogel matrices provide enhanced capability for drug testing in breast cancer (BCa), by better mimicking key physicochemical characteristics of this disease’s extracellular matrix. Here, we encapsulated patient-derived breast cancer cells in bioprinted polyethylene glycol-derived hydrogels (PEG), functionalized with adhesion peptides (RGD, GFOGER and DYIGSR) and gelatin-derived hydrogels (gelatin methacryloyl; GelMA and thiolated-gelatin crosslinked with PEG-4MAL; GelSH). Within ranges of BCa stiffnesses (1–6 kPa), GelMA, GelSH and PEG-based hydrogels successfully supported the growth and organoid formation of HR+,−/HER2+,− primary cancer cells for at least 2–3 weeks, with superior organoid formation within the GelSH biomaterial (up to 268% growth after 15 days). BCa organoids responded to doxorubicin, EP31670 and paclitaxel treatments with increased IC_50_ concentrations on organoids compared to 2D cultures, and highest IC_50_ for organoids in GelSH. Cell viability after doxorubicin treatment (1 µM) remained >2-fold higher in the 3D gels compared to 2D and doxorubicin/paclitaxel (both 5 µM) were ~2.75–3-fold less potent in GelSH compared to PEG hydrogels. The data demonstrate the potential of hydrogel matrices as easy-to-use and effective preclinical tools for therapy assessment in patient-derived breast cancer organoids.

## 1. Introduction

Cancer treatment is challenged by the multifaceted nature of cancer as a dynamic and heterogeneous disease, contributing to therapeutic resistance and treatment failures in patients [1]. Heterogeneity arises from genetic and epigenetic factors, extrinsic stromal factors and other dynamic factors, including cancer stage and treatment history, specific to each individual [2]. Initially, tumor cells modulate the tumor microenvironment, that is comprised of organ-specific extracellular matrices (ECMs), with the resulting activated stroma typically generating feedback loops that fuel the crosstalk between intrinsic and extrinsic factors, rendering each single tumor more unique over time [3,4]. In breast cancer (BCa), the most frequently diagnosed and lethal cancer among women worldwide [5], next-generation sequencing studies have identified more than 1600 driver mutations in over 90 breast cancer genes across 20 distinct subtypes of BCa [6], epitomizing the landscape of BCa heterogeneity. Yet, the standard anti-neoplastic drugs used to treat or improve survival of BCa patients are based on clinical and pathological features (such as hormone receptor (HR) and human epidermal growth factor receptor 2 (HER2) statuses). Even when the genetic landscape from the patient is available and integrated in the decision making via software technology, many predictions remain incorrect. This can result in the administration of unsuitable and ineffective treatments (over-treatment, delay in best treatment) and may lead to unnecessary toxic side-effects, prompting the need for individualized experimental testing [7].

In the last 15 years, three-dimensional (3D) organoid technologies have been proposed as innovative tools for therapeutic screening and precision medicine in cancer treatment [8,9]. Addressing the high genomic instability and altered phenotypes of 2D cultures, 3D organoid culture systems from patient specimens better retain the characteristics of a patient’s original tumor [10]. They provide enhanced tumor biomimetics owing to the complex cell-ECM interactions, which are crucial for assessing a patient’s sensitivity to treatment. Patient-derived organoids (PDOs) are also more reproducible than organotypic slices [1], can be perpetuated while retaining the mutational aberrations of parenteral tumors, and can be viably frozen for further use. PDOs also address the inefficient, time-consuming, and labor-intensive derivation of patient-derived xenografts (PDXs), which cannot realistically contribute to individualized therapy on a broad scale. Inspired by 3D culture of the mammary epithelium, BCa PDOs have been successfully generated with >100 primary and metastatic BCa organoid lines, fueling mechanistic research and drug development [11]. Yet, while PDOs represent a significant improvement in the biomimetic culture of primary cancer cells, the lack of tailorable ECMs in standard PDO culture has impaired targeted physicochemical manipulation of the extrinsic PDO microenvironments. The ability to tailor to such parameters will enable superior tumor biomimetics and extended PDO longevity ex vivo, necessary to assess the effects of recurring drug treatments or radiotherapies over longer periods of time [1].

Advances in biomaterials and biomanufacturing technologies are providing an increasingly rich toolbox that enables enhanced recapitulation of specific 3D microenvironments [12,13,14]. Synthetic and semi-synthetic 3D matrices offer tailored physicochemical cues (cell adhesion motifs, stiffness gradients, compartmentalized architectures, pH-responsive properties) [15], while inviting cells to deposit their own ECM rather than being cued to develop specific phenotypes, as seen with natural matrices, such as collagen [16] or fibrin [17]. However, despite increasing reports of relevant new strategies for drug screening utilizing 3D cell culture techniques, the majority of such reports use cell lines [18]. However, the gold standard for the 3D cell culture of patient-derived cells for applications in personalized medicine remains the natural biomaterial Matrigel^®^ [19].

Derived from basement membrane extract from a mouse tumor ECM, Matrigel^®^ enables rapid 3D cell encapsulation, yet lacks the ability for extensive biophysical manipulation [1] and compounds patient heterogeneity with its own batch-to-batch heterogeneity [20]. With stiffness ranges of 0.03–1 kPa (Young’s moduli) [21], Matrigel^®^ does not recapitulate the pathological stiffnesses found within BCa tissue. With increased matrix protein deposition and collagen-I crosslinking, changes in both tissue organization and composition during BCa establishment and progression lead to significant stiffness changes [22]. In BCa, Young’s moduli typically increase from 100–400 Pa for healthy tissue to 1–5 kPa [23,24] but can reach up to 42.5 kPa (high grade invasive ductal carcinoma) [25], depending on tumor type. Overall, tumor ECM stiffness is a critical aspect that drives progression and metastasis in BCa [22]. It is linked to BCa cancer subtype and is an indicator of disease progression outside the mammary compartment and metastatic potential [26]. Stiffness further informs response to therapy and overall prognosis, and as such is a critical component that needs addressing in organoid modelling [1].

The synthetic biomaterial polyethylene glycol (PEG, used in stiffness ranging from 1–50 kPa in BCa applications [26]) has been extensively used as a malleable alternative to Matrigel^®^ [27] and is favored due to the ability to add ECM proteins (cell adhesion and degradable ligands) that can support natural ECM deposition (GAGs, collagens) while decreasing biomaterial heterogeneity. Yet, fewer cases of PEG-based culture of patient-derived samples have been reported than Matrigel^®^. For example, PEG-based hydrogels functionalized with acrylate [28] and hyaluronan [29] have been used for the culture of patient-derived prostate cancer xenografts. Using BCa cell lines, PEG has been used copolymerized with maleimide, phosphocholine and polyacrylamide to compare MDA-MB-231 and SKBR3 cell lines for example, successfully comparing potency of cytotoxic agents and drug inhibitors [30]. An added advantage of PEG derivatives is its use in bioprinting [31], which may open the door to automated and medium-to-high-throughput manufacturing of BCa PDO models [32]. Yet, the limited bioactive sites and lack of protein absorption of PEG has prompted the field to explore semi-synthetic polymers [8].

Semi-synthetic gelatin-based hydrogels are promising alternative biomaterials that have been widely studied for 3D cell culture [33,34], as well as for BCa applications [8,20]. Arising from denatured collagen, the most abundant BCa ECM protein, gelatin can be functionalized with methacrylate groups to form GelMA, a biomaterial that combines the bioactivity of gelatin with the mechanical tuning provided by the photocrosslinkable methacryloyl functional groups [35]. GelMA has been used with BCa cell lines (MDA-MB-231, MDA-MB-468, MCF-7, SKBR3 [36,37,38]) showing how 3D cultures were more resistant to paclitaxel treatment [36], or how cell-imprinted GelMA hydrogels (topography-modified) led to enhanced doxorubicin uptake and increased cell death [37]. While GelMA provides platforms that are mechanically adjustable to match the normal and pathological breast environments, the use of UV crosslinking and photoinitiators may potentially harm primary cells, although this remains unaddressed [33].

Michael-type click-chemistry crosslinking of gelatin offers a potentially more cytocompatible way to provide gelatin-based hydrogels for the culture of sensitive primary breast cancer cells. Typically, mammalian sources (porcine and bovine) are used to produce gelatin-based 3D matrices, such as GelMA, due to an increase in proline and hydroxyproline amino acids compared to non-mammalian sources (piscine) leading to an increase in collagen-like triple-helix formation, and therefore, enhanced mechanical properties [36]. As such, mammalian-based gelatins possess high viscosity and shear-thinning flow behavior at room temperature, limiting their use for drop-on-demand bioprinting. Recently, we have demonstrated how fish gelatin could address this issue as it exhibits low viscosity and Newtonian-like flow behavior at room temperature [39]. Fish gelatin could be successfully thiolated using EDC/NHS L-Cysteine conjugation, to form Gel-SH, a biomaterial that can undergo Michael-type click crosslinking with 4-armed PEG-maleimide (PEG-4MAL) groups to form hydrogels near-instantaneously. MDA-MB-231 and MCF-7 BCa cell lines were successfully cultured up to 21 days in Gel-SH/PEG-4MAL hydrogels (referred hereafter as ‘GelSH’ hydrogels, for simplicity) [39], however, it is not yet known whether GelSH-based hydrogels can be applied to patient-derived BCa cells, which will be the purpose of this study.

The aim of this work is to provide a direct comparison of three hydrogel biomaterials to support patient-derived BCa ex vivo organoid cultures to be used as drug testing platforms. Within ranges of BCa stiffnesses, GelMA, GelSH and PEG-based hydrogels of 1–6 kPa stiffnesses successfully supported the growth and organoid formation of HR+,−/HER2+,− primary cancer cells at least to 2–3 weeks, with superior organoid formation within the GelSH biomaterial. BCa PDOs responded to doxorubicin, EP31670 and paclitaxel treatments with increased IC_50_ on organoids obtained with the GelSH material. These data demonstrate the potential of synthetic and semi-synthetic hydrogel matrices as easy-to-use and effective preclinical tools for therapy assessment in patient-derived breast cancer organoids supported by relevant ECMs.

## 2. Materials and Methods

### 2.1. Tumor Tissue Collection

Patients consented use of their tissues following admission for breast cancer surgery at the breast cancer unit either at the Princess Alexandra Hospital or Mater Hospital, Brisbane, Australia. The study was performed in accordance with the principles of the Declaration of Helsinki and within ethical and institutional guidelines (ethics number HREC/2020/QRBW/61294 and HREC/17/MHS/50, approved 28 July 2017), and in accordance with the standards and protocols of the Centre for Personalised Analysis of Cancers (CPAC, Brisbane, Australia) and the Mater-Queensland University of Technology (QUT) Breast Cancer Research Biobank [40]. Four primary patient-derived breast cancer tumor materials were used in this study (Table 1); three directly from primary breast cancer (BCa) patients undergoing surgery and one from a mouse xenograft. The three primary BCa tissues, surplus to pathology needs, were collected from consented donors during breast cancer surgery (two samples were progesterone positive, estrogen positive and human epidermal growth factor 2 negative (ER+/PR+/HER2− subtype, P#03F and P#04F) and one sample was from the ER−/PR−/HER2+ subtype (P#01F). The patient-derived xenograft (PDX) sample (P#02X) was generated by implanting a 50 mm^3^ piece of a freshly excised triple-negative (ER−/PR−/HER2−) breast tumor into the left inguinal mammary fat pad of a 6-week-old female NOD/SCID mouse. Once tumor size reached ~800 mm^3^, PDX was passaged once using the same procedure before the mouse was terminated and the tumor excised for processing (Passage 2). All samples were transferred under sterile conditions and processed in PC2 laboratories and biosafety hoods.

### 2.2. Tumor Tissue Processing and Culture

Surgically-resected tumor specimens were placed in a sterile Petri dish, minced to 0.5 × 1 mm^3^ fragments using a sterile surgical scalpel and transferred to a sterile 50 mL centrifuge tube. A volume of 1 mL of 10× hyaluronase/collagenase (STEMCELL technologies, Tullamarine, VIC, Australia) and 4 mL of organoid media (ORM) [41] was added. ORM consisted of the following components supplied from Thermofisher, Brisbane, QLD, Australia; advanced DMEM/F12 (Gibco™, Waltham, MA, USA), supplemented with 10% (*v*/*v*) fetal bovine serum (FBS), 1% (*v*/*v*) penicillin streptomycin (P/S), HEPES 1M, GlutaMax 1×, Epidermal Growth Factor (EGF, 10 ng/mL). Y-27632 (5 µM) was purchased from Abmole, Australia and Hydrocortisone (1 µg/mL) from Sigma, Macquarie Park, NSW, Australia. After one to two hours of digestion, the samples were filtered through pre-wet reversible 100 µm and 37 µm strainers (STEMCELL Technologies, Tullamarine, VIC, Australia) into a new 50 mL centrifuge tube to remove large insoluble material [40]. While undigested material was kept and cultured with ORM in T75 flasks (Corning^®^, Mulgrave, VIC, Australia), the cell eluates obtained from the 37 µm strainers were diluted in Dulbecco’s Phosphate Buffered Saline (DPBS, Thermofisher) and used to assess cell viability upon digestion using Trypan Blue (Thermofisher) and automatic cell counter (Bio-Rad-TC20^TM^, South Granville, NSW, Australia). Cells were cultured in ORM with full media changes twice a week until reaching 80% confluency and being lifted using prewarmed 0.25% (*v*/*v*) Trypsin/ethylenediaminetetraacetic acid (EDTA, Thermofisher) in DPBS.

### 2.3. Cell Encapsulation in Bioprinted PEG Hydrogels

Cells were printed using a non-contact drop-on-demand 3D bioprinter (RASTRUM, Inventia Life Science, Alexandria, NSW, Australia), as previously described [42]. We used polyethylene bioink formulations of 1 or 3 kPa stiffness with peptides motifs, including Arg-Gly-Asp (RGD), Asp-Tyr-Ile-Gly-Ser-Arg (DYIGSR) and Gly-Phe-Hyp-Gly-Glu-Arg (GFOGER.) The bioinks contained either RGD (Cat. P×02.31P), RGD + GFOGER (Cat. P×2.09P), or RGD + GFOGER + DYIGSR (Cat. P×02.28P and P×03.28P) and were supplied from Inventia Life Science. The structure designs and printing protocols were first created using RASTRUM Cloud (Inventia Life Science). We used the 3D Imaging Model (5 × 10^6^ cells/mL input concentration, 1600 cells/well after printing) and 3D Large Plug Model (5 × 10^6^ cells/mL input concentration, 14,400 cells/well after printing) according to the manufacturer’s protocol in a two-stage process using bioinks and activators. Briefly, the bioinks were loaded in the cartridge and printed on a 96-well-plate using multiple independently addressable microvalves, which eject fluids at nanolitre precision [43]. In the meantime, the cells were trypsinised, counted and resuspended in the activator solution in the biosafety hood. Next, the cell-containing activator solutions were loaded in the bioprinter cartridge and bioprinted onto the bioink-containing wells of the well-plate, forming instantaneous 3D hydrogels by click-chemistry (Michael-Type addition). Upon completion of the printruns, 150 µL ORM was added to each well and the plate was incubated at 37 °C until further analysis and drug treatments.

### 2.4. Cell Encapsulation in Photocrosslinkable GelMA Hydrogels

Gelatin methacrylamide (GelMA) stock solutions (15% *w*/*v* porcine Type A, 80% degree of functionalisation, 300 bloom, in DPBS) were purchased from Gelomics Pty Ltd.™, Kelvin Grove, QLD, Australia, and processed as described previously [44]. Briefly, GelMA hydrogel precursor solutions were prepared by diluting the 15% (*w*/*v*) stock solution with DPBS to 4% (*v*/*v*) and contained 0.05% (*w*/*v*) of the photoinitiator Irgacure 2959 (1-[4-(2-hydroxyethoxy)-phenyl]-2-hydroxy-2-methyl-1-propanone, BASF, Ludwigshafen, Germany). Precursor solutions were incubated at 37 °C until use. Custom polytetrafluoroethylene (PTFE) casting molds (5 mm diameter, 2 mm depth, QUT Design and Manufacturing Centre, Brisbane, QLD, Australia) were sterilized with 80% (*v*/*v*) ethanol and exposed to UV light for 20 min in a biosafety hood. Prewarmed precursor solutions containing the cells (2 million cells/mL) were dispended into the mold, covered by a glass slide and crosslinked for 15 min (365 nm, 2.6 mW/cm^2^ intensity, reaching a final stiffness of 3 kPa [44]) using a UV crosslinker (CL-1000 L, UVP, Upland, CA, USA). Using a sterile spatula, gels were transferred from the glass slide to a well-plate and washed with DPBS for 15 min. ORM (400 µL) was then added to each well and the plate was incubated in a humidified incubator (37 °C, 95% air, 5% CO_2_) until further analysis and drug treatments.

### 2.5. Cell Encapsulation in Click-GelSH Hydrogels

GelSH hydrogel precursor solutions were prepared according to [39]. Briefly, 10 g gelatin from cold-water fish skin (Sigma, St. Louis, MO, USA) was added to 500 mL 0.1 mM HCl and stirred at room temperature (RT) until dissolved. Next, 7.5 g 1-ethyl-3(3-dimethylamino)propyl carbodiimide (EDC, Sigma) and 3.75 g *N*-hydroxysuccinimide (NHS, Sigma) were added and stirred for 30 min, followed by addition of 20 g L-cysteine (Sigma) to the solution. After 24 h, the solution was dialyzed against 0.1 mM HCl using 1 kDa molecular weight cut-off dialysis tubing (Sigma) for 5 days. Once dialysis was complete, samples were frozen overnight at −80 °C and lyophilized for 5 days. The lyophilized Gel-SH powder was then dissolved in 300 mM HEPES (Thermofisher) to stock concentrations of 20% (*wt*/*v*), 10% (*wt*/*v*) and 5% (*wt*/*v*) and kept on ice. Equimolar PEG-4MAL hydrogel precursor solutions of similar concentrations were prepared by dissolving PEG-4MAL (MW 20 kDa, JenKem Technology, Plano, TX, USA) in 300 mM HEPES (Thermofisher) and kept on ice. A volume of 10 µL of PEG-4MAL was added to the wells of 48 well-plates. Breast cancer cells were then resuspended in Gel-SH (4 × 10^6^ cells/mL input concentration) and 10 µL of the cell suspension was pipette-mixed within each PEG-4MAL drop until crosslinking occurred (<3 s) for a final concentration of 2 million/mL in hydrogels of final concentrations of 2.5%, 5% and 10% (*w*/*v*), corresponding to 1, 3 and 6 kPa stiffness [39]. ORM (300 µL) was then added to each well and the plate was incubated at 37 °C until further analysis and drug treatments. Hereafter, the resulting GelSH/PEG-4MAL hydrogels are referred as to the ‘GelSH’ hydrogels. No comparison to Matrigel was done in this study, due partly to worldwide shortage at the time of the study, but also due to the inability to know exactly what elastic modulus (Young) the supplied batch would have been, and likely lower than 1 kPa. This is lower than most BCa tumors (hence not relevant) but also below what can be mimicked with the other gel systems presented in this study. Unable to match stiffnesses, nor decouple whether results would be due to differences in ECM composition or in stiffnesses, a comparison would have been controversial.

### 2.6. Cell Viability 

Viability was assessed at day 2 and day 12 post hydrogel encapsulation using fluorescein diacetate (FDA, Thermofisher) and propidium iodide (PI, Thermofisher). Cell media was aspirated, and cell-laden hydrogels were washed with DPBS at room temperature for 5 min, then incubated with staining solution (0.67 μg/mL FDA, 5 μg/mL PI and 5 μg/mL Hoechst (Thermofisher) in DPBS) for 10 min. The staining solution was aspirated, and samples were washed for 5 min in DPBS, twice. The samples were immediately imaged using an inverted epifluorescence microscope (IX73 Olympus, Atlanta, GA, USA) equipped with either a DP72 camera or XM10 camera, set on the red (excitation 561 nm), green (excitation 488 nm) and blue (excitation 405 nm) filter sets to assess live cells, dead cells and cell nuclei, respectively, with matched brightfield acquisition.

### 2.7. Metabolic Activity 

At specific time points upon normal culture and following drug treatments, metabolic activity was measured using the PrestoBlue cell viability assay (Thermofisher, Waltham, MA, USA). 3D hydrogels were incubated with ORM, containing 10% (*v*/*v*) PrestoBlue cell viability assay for three hours in a humidified incubator. Cell-free gels with the corresponding media were used as negative controls. Upon incubation, the solutions were transferred in duplicate-triplicate (90 µL) into 96-well plates (Corning^®^). Fluorescence (excitation 544 nm, emission 590 nm) was determined using a FLUOstar Omega plate reader (BMG Labtech, Ortenberg, Germany) and corrected with negative control background.

### 2.8. Immunofluorescence Staining and Imaging 

Upon culture, the media was aspirated and washed with DPBS twice. The hydrogels were fixed in 4% paraformaldehyde (PFA, Sigma) for 30 min at RT, and washed in DPBS two times (10 min each), before permeabilization in 0.2 % (*v*/*v*) Triton X-100 in DPBS for 20 min at RT. After two washes in PBS (5 min each), samples were treated with 1% (*w*/*v*) bovine serum albumin (BSA) in PBS for 15 min, followed by 5% (*w*/*v*) BSA in PBS for 2 h. Primary antibody solutions in 1% BSA/PBS (mouse anti-human pan-Cytokeratin, ab215838, Abcam (Cambridge, UK), 1:50; mouse anti-human vimentin, eFluor^TM^ 660, eBioscience (Thermofisher, Waltham, MA, USA), and mouse anti-human Anti-gamma H2A.X, ab26350, Abcam, 1:500), were used in 2D or on the hydrogels (50–200 µL/hydrogel) overnight at 4 °C on gentle shaking. After two PBS washes (10 min each) and an overnight wash in PBS at 4 °C on gentle shaking, the samples were incubated with secondary antibody (Goat anti-mouse IgG AlexaFluor555 preabsorbed, ab150118, Abcam, 1:200) in 1% (*w*/*v*) BSA/PBS, containing FITC-conjugated phalloidin (1:200, 200 U/mL, Thermofisher) and DAPI (1:1000, 5 µg/mL, Sigma) overnight at 4 °C on gentle shaking. After two washes in PBS (10 min each) and an overnight wash in PBS at 4 °C on gentle shaking, fresh PBS was added. The plate was covered by foil and kept at 4 °C until imaging. 3D imaging was done using a Nikon spectral spinning disc confocal microscope (SDC, X-1 Yokogawa spinning disc with Borealis modification) fitted with either a Plan Apo 10× or Plan Fluor ELWD 20× DIC objectives. Specific z-stack details are reported individually for each characterization. Z-stacks of hydrogels were captured with 2.5 μm step size, and maximum intensity projections of hydrogel Z-stacks were obtained using the ImageJ software (version 1.52a, National Institute of Health (NIH), Bethesda, MD, USA) [45].

### 2.9. Drug Treatments 

Patient-derived cells grown in 2D and in 3D organoids in hydrogels were treated with doxorubicin hydrochloride (Sigma), paclitaxel (Sigma) and EP31670 (A gift from Professor Claes Wahlestedt, University of Miami Miller School of Medicine, Miami, FL, USA). Serial dilutions were prepared from stock solutions of doxorubicin 10 mM (dissolved in DMSO, Sigma) to a 1–100 µM final range, of paclitaxel 5 mM (used as supplied) to a 1–100 µM final range and 1 mM EP31670 (dissolved in DMSO) to a 0.05–10 µM final range, using ORM. After 3D encapsulation and satisfactory organoid structures above 50 µm in diameter (8 days on average), drugs were administered to the PDO cultures, including DMSO vehicle control. Media containing fresh drugs was changed every 3–4 days, which was done 2 and 3 times depending on the experiments (refer to figure timelines).

### 2.10. Cell Cytotoxicity Assays 

The metabolic activity of 2D and 3D patient-derived cells upon drug treatments was assessed by the CellTiter-Glo^®^ cell viability reagent (Promega, Alexandria, NSW, Australia) according to the manufacturer’s instructions. Briefly, the diluted CellTiter Glo substrate (50 µL) was added to the culture wells at the end of the treatment period, followed by a 30 min incubation on a shaker at RT. The substrate was aspirated and plated (2–3 replicates of 100 µL) in new 96 well-plates. Luminescence was quantified using a FLUOstar Omega plate reader (BMG Labtech, Offenburg, Germany). IC_50_ curves were determined by GraphPad software using the non-linear regression analysis, log(inhibitor) vs response, variable slope (four parameters) mode.

### 2.11. Statistical Analysis 

All statistical tests were performed in IBM SPSS Statistics 23 (IBM Corp). For comparison between 2 groups and *n* < 4/group, Mann-Whitney non-parametric test was done on medians. When more than two groups were compared, 1-way ANOVA was used with Tukey Post hoc test. For more than two groups and more than two variables, a general linear model (univariate analysis) was used with Tukey Post hoc test, performed when overall significance was met.

## 3. Results and Discussion

### 3.1. Assessment of 3D Hydrogel-Based Biomaterials for BCa PDOs

**Figure 1:**
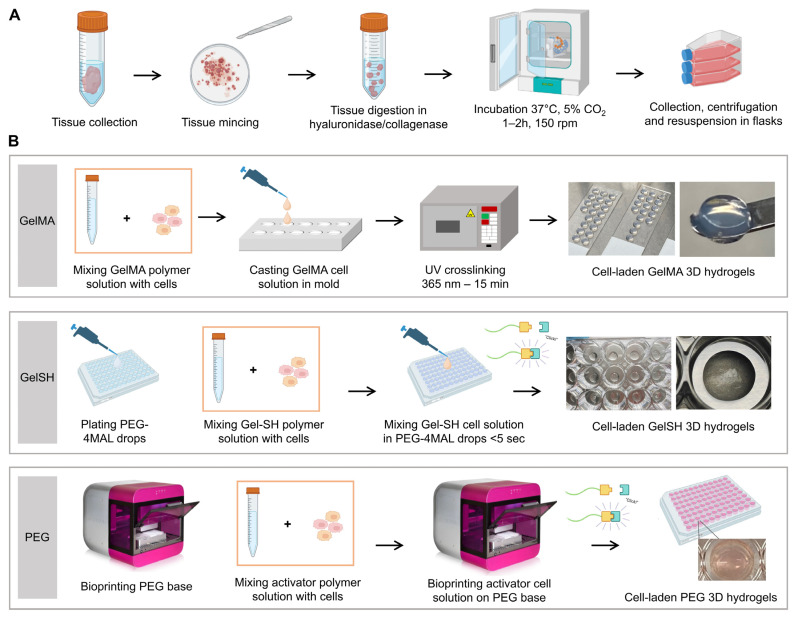
Schematic representation of tissue processing from tumor collection and preparation (**A**) to encapsulation into 3D hydrogels (**B**). GelMA = gelatin methacryloyl. GelSH = Gel-SH/PEG-4MAL, PEG = polyethylene glycol. Figure partly created with BioRender.com.

In this study, we assessed the ability of various hydrogel-based biomaterials to support the 3D organoid formation of patient-derived breast cancer (BCa) cells derived from patient tissue following tumor resection, surplus to pathology needs. The tumor pieces were successfully reduced to individual cells, according to established protocols [40]. Relying on tissue mincing, enzymatic digestion and mechanical agitation, cells were then cultured and expanded in 2D (Figure 1A) using a suitable media validated for human BCa-derived organoids [41]. While it is acknowledged that this may lead to specific cell populations being selected, this strategy is needed to obtain sufficient cell numbers prior to 3D printing, as the surplus to pathology needs received for research purposes is too small. The specific organoid media we used was mostly similar to the medium by Guillen et al. and is recommended for BCa organoids [41]. It contains serum and specific growth factors, such as epidermal growth factor (EGF), that enhance epithelial cell proliferation, as well as the Rho kinase inhibitor (Y-27632), a critical supplement to support BCa ex vivo cultures past 15 days [41].

The encapsulation of the patient-derived cells in three different types of hydrogels is highlighted in Figure 1B, referred to as PEG, GelMA and GelSH hydrogels. Table 2 highlights some differences between these hydrogels. While PEG and GelSH are instantaneously crosslinked via Michael-type addition, GelMA requires the addition of a photoinitiator in the precursor solution and UV light for crosslinking. Despite using the same chemistry, compared to the manual crosslinking for GelSH, bioprinting enabled the PEG gels to be manufactured in an automated way at nanoliter precision [42], generating smaller volumes of hydrogels. The resulting gels could be made in different volumes, typically used in ranges from 0.8 to 60 µL, which could be tailored to contain 640 to 130,000 cells/gel. Such tailoring is advantageous when patient material for research purposes is limited [1].

#### 3.1.1. Effect of Peptide Functionalization in Bioprinted PEG-Derived Hydrogels

**Figure 2:**
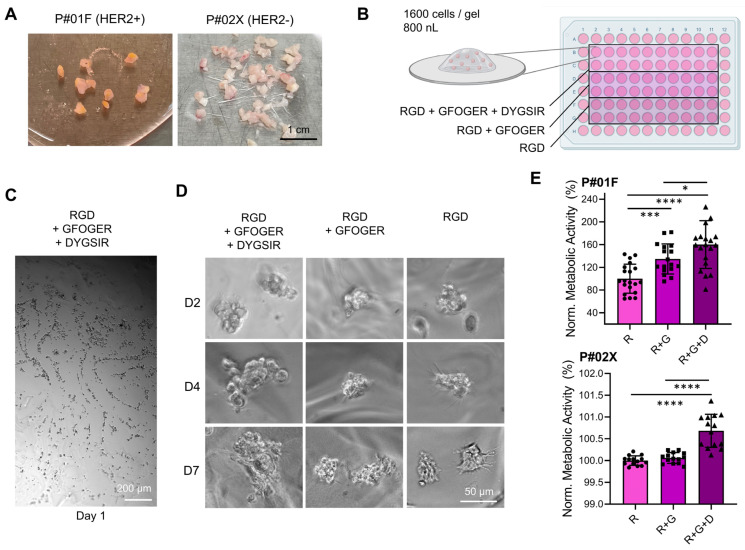
Effect of peptide functionalization on breast cancer patient-derived tumor tissues digested and bioprinted as individual cells in PEG hydrogels (P#01F and P#02X). (**A**) Photographs of tumor tissues upon excision. (**B**) Schematic of bioprinted well-plate conditions. (**C**) Overview of single cell distribution in PEG hydrogel containing RGD + GFOGER + DYIGSR one day after single cell bioprinting (P#02X). (**D**) Brightfield images of cells in PEG hydrogels functionalized with different peptides over time, showing enhanced organoid formation for the PEG-(RGD + GFOGER + DYIGSR) formulation (P#01F). (**E**) Metabolic activity after 7 days of culture, normalized to the PEG with RGD group (R) for each tumor (R = RGD, G = GFOGER, D = DYIGSR). Circles, squares, and triangles show individual datapoints. Histograms show means ± SD, *n* = 17–20 technical replicates (* *p* < 0.01, *** *p* < 0.001, **** *p* < 0.0001).

The inclusion of adhesion ligands is critical to the success of PEG hydrogels as 3D cell culture models [46] biomimicking the intricate tumor microenvironment and its ECM, which comprises a multitude of fibrous proteins, matricellular-associated proteins, and proteoglycans [47]. Conjugation of specific adhesion peptides such as the integrin-binding motif RGD (Arg-Gly-Asp), laminin fragment peptide DYIGSR (Asp-Tyr-Ile-Gly-Ser-Arg) and collagen-1 sequence GFOGER (Gly-Phe-Hyp-Gly-Glu-Arg) gives an opportunity to control cell adhesion and integrin activation and cell-matrix interactions [48], enabling us to study the role of specific ligands in the process of organoid formation [42]. Here, we used a fresh patient tumor and a xenograft tumor (Figure 2A, patient P#01F and P#02X) in PEG hydrogels containing various combinations of these three peptides at 1 kPa stiffness (Figure 2B). Automated bioprinting here enabled the use of a ‘reductionist’ 3D cancer model with a final hydrogel volume of 2880 nL/well containing 1600 cells/gel. Cell distribution following printing was homogeneous (Figure 2C) and organoids formed over time, although this was minimal for RGD only or RGD + GFOGER, but best for RGD + GFOGER + DYIGSR (Figure 2D). This suggests that the addition of DYIGSR peptide in PEG-based hydrogels is more critical than RGD or RGD + GFOGER to ensure organoid growth. After 7 days of culture, the resulting organoids with the latter formulation were larger (Figure 2D) and had higher metabolic activity (Figure 2E). This is in line with previous literature where peptide combination was more effective for organoid growth than their individual counterparts, due to higher number of adhesions sites [49]. For instance, incorporation of YGSIR in PEG hydrogels limited adhesion and no spreading of human microvascular endothelial cells and vascular smooth muscle cells, which was strongly enhanced when YGSIR was combined with RGD [41]. This suggests that desired response of specific cell types to PEG hydrogels could be optimized through the combinatory use of biomimetic peptides. This was also applied to the investigation of ER + BCa dormancy which was better mimicked using PEG hydrogels functionalized with GFOGER and IKVAV from the collagen I and laminin α1 chain, respectively, by forming protein-polysaccharide complexes for cell adhesion [50].

#### 3.1.2. Effect of Stiffness in Bioprinted PEG-Derived Hydrogels

**Figure 3:**
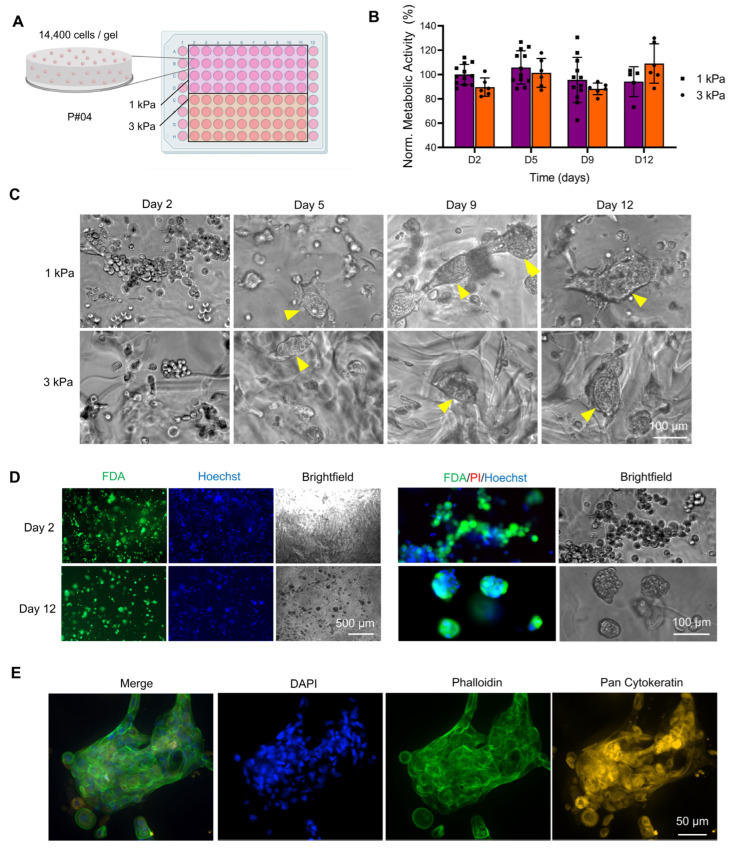
Effect of PEG-based hydrogel stiffness on growth and viability of breast cancer patient-derived organoids (P#04F). (**A**) Schematic of bioprinted well-plate conditions, comparing 1 and 3 kPa PEG hydrogels functionalized with RGD + GFOGER + DYIGSR. (**B**) Metabolic activity over time, normalized to the 1 kPa group at Day 2. Means ± SD shown, *n* = 6–12 technical replicates. No statistical differences. (**C**) Brightfield images of cells in hydrogels over time, showing organoid formation (yellow arrows) after 5 days of culture for both stiffnesses. (**D**) Epifluorescence images of FDA/PI/Hoechst staining and corresponding brightfield microscopy images, showing live cells (green), dead cells (red), cell nuclei (blue), and overall morphology, respectively, at 2 and 12 days post-bioprinting for 3 kPa PEG hydrogels. (**E**) Confocal fluorescence images of immunofluorescence for cell nuclei (blue), cytokeratin (Pan-CK, yellow), F-actin (green) after 12 days of culture for 3 kPa hydrogels.

BCa stiffnesses vary mostly between 1 and 5 kPa, although higher stiffness can be observed [7,25,51]. Assessment of the effect of PEG stiffness was performed here with hydrogels of 1 kPa and 3 kPa stiffnesses using the optimized PEG formulation supplemented with RGD + GFOGER + DYIGSR (Figure 3A). The metabolic activity of cells was constant over 12 days of culture with no significant differences between the two stiffnesses (Figure 3B), yet the cells formed cohesive 3D structures that were irregular and complex (Figure 3C). This is in line with MCF7 cell cultures in PEG hydrogels printed with the same platform, supplemented with RGD, which remained more round when cultured in the RGD-free counterpart [32]. Viability was maintained throughout the 12 days of culture (Figure 3D), with a final metabolic activity of 94 ± 14% and 109 ± 14% for 1 and 3kPa, respectively (*p* > 0.05, no statistical differences). No necrotic core was observed within this timeframe in line with the fact that only larger spheroids (>200 µm) can form oxygen and nutrient gradients which lead to the formation of proliferation gradients and zonal heterogeneity [52]. The absence of a necrotic core is useful to isolate the effects of 3D geometry and elastic modulus, as seen with SK-BR-3 cells cultured PEG gels, shown to be more sensitive to Sorafenib, but not Lapatinib, due to the fabricated ECM, platform geometry and stiffness [53]. Finally Figure 3E shows how most of the organoids displayed cytokeratin staining, validating epithelial origin of the patient-derived cells cultured here.

#### 3.1.3. Effect of Hydrogel Type (PEG, GelMA, GelSH)

**Figure 4:**
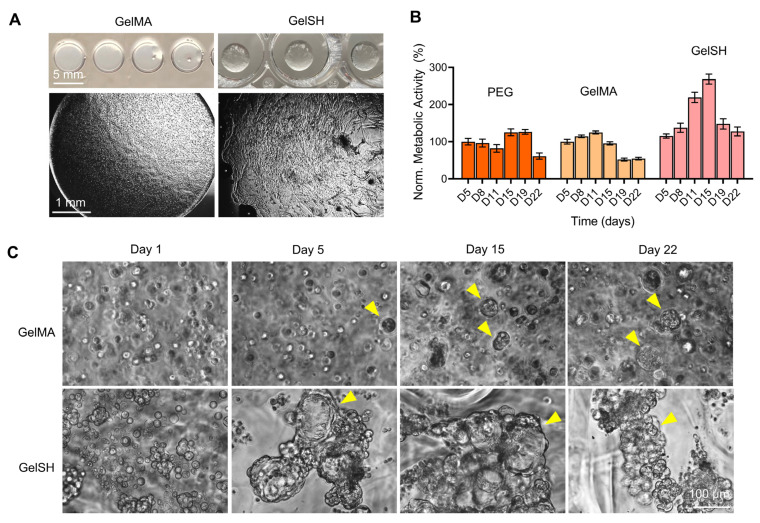
Effect of gel type on the growth and viability of breast cancer patient-derived organoids (BCa PDOs) for similar gel stiffness (3 kPa) up to 22 days of culture (P#03F). (**A**) Macroscopy and brightfield microscopy images of gelatin-based hydrogels; UV-crosslinked gelatin methacryloyl gels (GelMA) and click-crosslinked thiolated gelatin-based hydrogels (GelSH). (**B**) Metabolic activity of BCa PDOs over time, normalized to bioprinted PEG-(RGD + GFOGER + DYIGSR) group at Day 5. Means ± SEM shown, *n* = 4–16 technical replicates. (**C**) Brightfield images of cells in GelMA and GelSH gels over time, showing organoid formation (yellow arrows) after 5 days of culture for both gel types.

Based on previous results, the PEG formulation comprising RGD, GFOGER and DYIGSR at a 3 kPa stiffness was deemed optimal for the culture of BCa PDOs and selected for a comparison with two other types of gels at similar stiffness; the widely characterized gelatin-methacryloyl gel (GelMA, UV crosslinked) [8,33,34,36,54,55] and a novel thiolated gelatin-based hydrogel (GelSH, click-chemistry crosslinked) [39]. We sought to extend culture time to three weeks and observe whether there would be a limit to organoid formation and metabolic activity. First, Figure 4A shows how the macroscopic morphology of gelatin-based biomaterials looked following encapsulation, displaying homogeneous cell distribution. The metabolic activity in PEG and GelMA looked similar and constant for the first 15 days of culture, but then started to drop at day 22 for PEG and at day 19 for GelMA (Figure 4B). Conversely, metabolic activity in GelSH increased steadily during the first 15 days of culture and peaked at day 15, with 268 ± 33% growth compared to day 5 (only 125 ± 27% and 95 ± 11% for PEG and GelMA, respectively), but then started to decrease at day 19, in line with decrease in ATP levels after 15 days [43]. This decrease in metabolic activity coincided with morphological changes to organoids which from day 19 onward in GelSH (Figure 4C and Figure S1). Morphology images showed enhanced organoid formation in the GelSH group compared to GelMA over time. BCa organoids in GelMA were smaller and more compact compared to organoids in GelSH and only isolated spheroids kept increasing over time, while smaller ones or individual cells did not. It is hypothesized here that the UV crosslinking affected cell growth of primary cells. This is line with previous reports which showed that UV light can affect certain cell types during GelMA crosslinking, as seen by decreased viability in UV-crosslinked GelMA containing KUSA-A1 cells, compared to visible-light-crosslinked GelMA [56], as well GelMA containing multipotent stromal cell (MSCs) [56] and odontoblast-like cells (OD21) [57], although it must be noted that crosslinking times and intensities were different. As for reduced metabolic activities between PEG and GelSH which were crosslinked using the same click-chemistry and at the same stiffness, it is hypothesized that the PEG does not sufficiently provide the ECM cues for the cells to continue their expansion journey compared to GelSH. Overall, it is noted that PEG and GelSH (click-chemistry in < 2 s) led to wavy organoid morphologies. This is likely due to gelation occurring faster than mixing. This results in softer stiffness pockets which create unintentional patterning for organoids to grow. This may be a potential issue for model reproducibility and can be addressed by varying the HEPES pH solution [39] for GelSH, so that crosslinking occurs in the order of min (instead of seconds) to ensure proper mixing prior to gelation.

#### 3.1.4. Effect of Stiffness in GelSH Hydrogels

**Figure 5:**
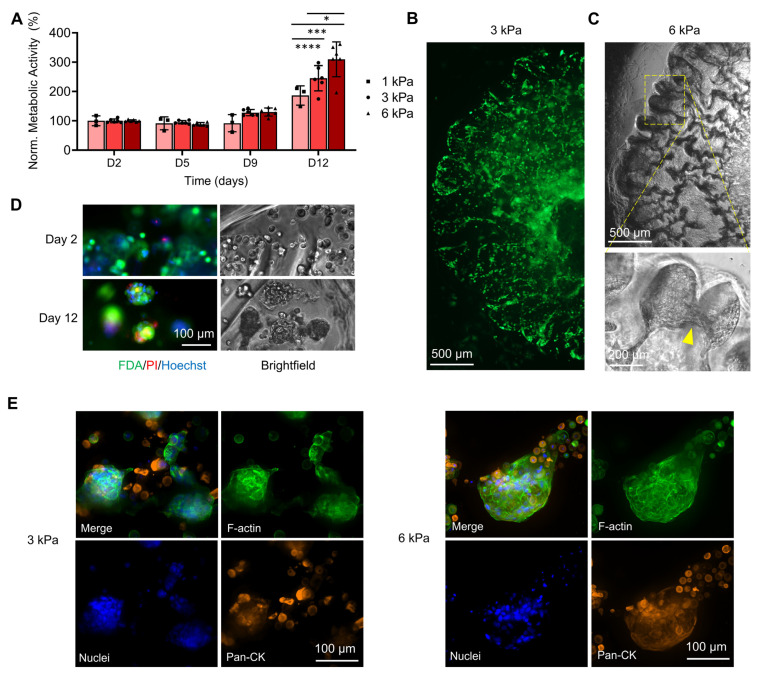
Effect of GelSH gel stiffness on the growth and viability of breast cancer patient-derived organoids (BCa PDOs) up to 12 days of culture (P#04F): (**A**) Metabolic activity of BCa PDOs over time, normalized to 1 kPa, Day 2. Means ± SD shown, *n* = 3–6 technical replicates (* *p* < 0.01, *** *p* < 0.001, **** *p* < 0.0001). (**B**) FDA staining of 3 kPa GelSH after 12 days of culture. (**C**) Brightfield images of cells in 6 kPa GelSH after 12 days of culture forming bridging organoids (yellow arrows). (**D**) Epifluorescence images of FDA/PI/Hoechst staining in 3 kPa GelSH and corresponding brightfield microscopy images, showing live cells (green), dead cells (red), cell nuclei (blue), and overall morphology, respectively, after 2 and 12 days of culture. (**E**) Confocal fluorescence images of immunofluorescence for cell nuclei (blue), cytokeratin (Pan-CK, orange), F-actin (green) after 12 days of culture.

The effect of stiffness on patient-derived cells was assessed in GelSH hydrogels of stiffnesses varying from 1 to 6 kPa (1, 3, 6 kPa, Figure 5). Morphologically (Supplementary Figure S2) and metabolically (Figure 5A), no differences were seen initially. The cells migrated and formed networks which densified over time. At 12 days, metabolic activity was correlated with stiffness increase (186 ± 33%, 245 ± 43% and 310 ± 66% for 1, 3, and 6 kPa respectively, normalized to Day 2), in line with the brightfield images which showed stronger network connections according to increasing stiffness (Supplementary Figures S2 and S3) and larger spheroid formation (Figure 5E). Additionally, all groups had high viability after 12 days, with only the 1 kPa group showing about 5% cell death (Figure 5B,D and Figure S3) which correlated with lower metabolic activity compared to the 3 kPa and 6 kPa groups. This is explained by a reduced number of RGD sites that are essential for cell adhesion at this stiffness, as seen previously in gelatin-based gels containing ovarian cancer cells that were below 1 kPa [58], and which can compromise initial cell attachment if insufficient.

### 3.2. Drug Response of 2D and 3D Hydrogel-Supported BCa PDOs

#### 3.2.1. Doxorubicin Response of 2D, Matrix-Free 3D, and Gelatin-Based 3D BCa PDOs

**Figure 6:**
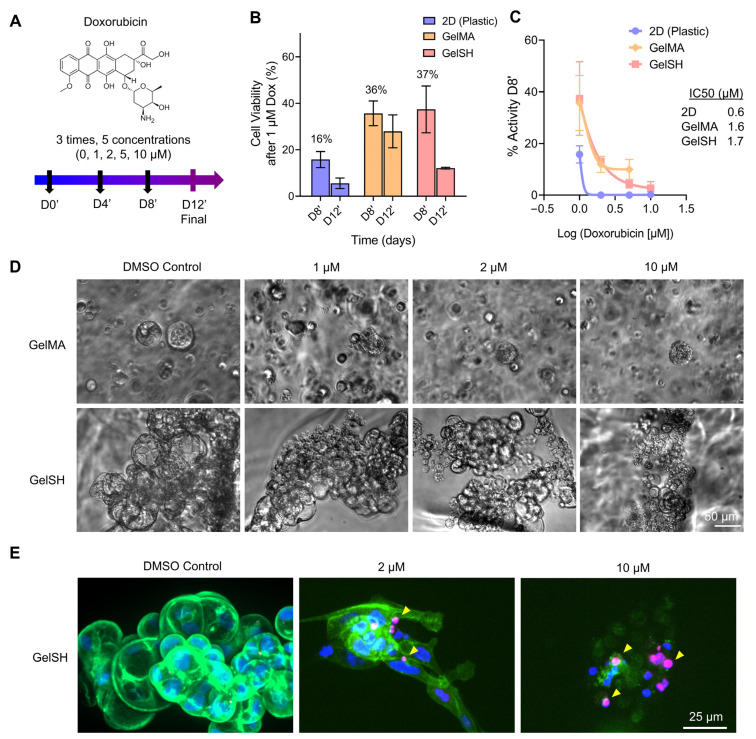
Doxorubicin response in 2D and for 3D PDO culture in gelatin-based biomaterials (GelSH and GelMA) of similar stiffness (3 kPa) from patient-derived cells (P#03F). (**A**) Doxorubicin treatment schedule. (**B**) Cell viability in 2D, GelSH and GelMA, following 1 µM doxorubicin treatment (Means ± SE, *n* = 3–4 technical replicates). (**C**) IC_50_ dose responses (Means ± SE, *n* = 3–4 technical replicates). (**D**) Brightfield images of entire 3D PDO morphology in GelSH and GelMA gels after 4 days of treatment. (**E**) Confocal images of immunofluorescence for DAPI (blue), H2Aγ (magenta), Phalloidin (green) staining showing local DNA damage (yellow arrows) after 12 days of drug treatment.

After validating the various hydrogels as 3D matrices for BCa PDO growth up to 15 days, we treated the gelatin-based 3D PDOs and corresponding 2D monolayers with a known standard-of-care model chemotherapy for BCa, doxorubicin, whereby ECM proteins contribute to mechanisms of drug resistance (Figure 6) [59]. First, we assessed drug response in patient-derived cells (P#04F) in 2D monolayers and matrix-free 3D configurations (Supplementary Figure S4). In 2D, total cell death was not observed after 4 days of treatment, even at 10 µM Doxorubicin (13 ± 2% viability), but this was achieved after 8 and 12 days of treatment (Supplementary Figure S4). This suggested that the latter two timelines (8 and 12 days of treatment) may be more relevant than the shorter timeline (3 days of treatment), especially when moving to 3D, where less drug potency would be expected [53]. The IC_50_ value, as measured after 4 days of drug treatment, was correspondingly higher (1 µM), compared to 8 days (0.6 µM) and 12 days (0.5 µM) of doxorubicin treatment (Supplementary Figure S4B). We then validated that the patient cells self-formed 3D organoids using Agar 1% (Supplementary Figure S4C) and responded to doxorubicin treatment (Supplementary Figure S4D,E). IC_50_ doses showed 3-fold reduced doxorubicin potency in 3D versus 2D, although a higher variability was noted with 3D cultures. This is expected when organoids of several sizes are generated, instead of one large organoid. The latter configuration can be achieved with round-bottom 384 plates, and will enable improved reproducibility [1].

We then sought to perform repeated drug treatments on the PDOs encapsulated in hydrogels, as we hypothesized that 3D cultures would require more sustained therapy. We thus monitored PDO morphology over time and analyzed drug response and resistance after 8 and 12 days of doxorubicin treatment. As could be expected from increased growth of PDOs in GelSH compared to GelMA (presented in Figure 4), PDO size at treatment start was larger in GelSH compared to GelMA (Figure 6D). However, PDOs in both GelSH and GelMA started to show partial organoid disintegration with as little as 1 µM doxorubicin and as early as 4 days (Figure 6D), which was also observed at day 8 post-treatment (Supplementary Figure S5). Increased doses correlated with further organoid collapse and cell death (Figure 6E), as evidenced by increased levels of DNA damage repair via γH2AX staining. γH2AX is a variant histone required for checkpoint-mediated cell cycle arrest and DNA repair following DNA lesions [60]. It is also required for DNA fragmentation during apoptosis and is phosphorylated by various kinases in response to apoptotic signals [61], representing an ideal marker to study drug response. Here the γH2AX foci increased over time, denotating more DNA damage with increasing doxorubicin concentrations. It was nevertheless shown that the viability of patient-derived cells was > 2-fold higher in 3D compared to 2D (16 ± 7% for 2D vs 36 ± 11% and 37 ± 14% for PDOs in GelMA and GelSH, respectively) after 8 days of 1 µM treatment (Figure 6B). The corresponding IC_50_ doses were similar between PDOs in GelMA and GelSH and exhibited ~2.75-fold reduced doxorubicin potency compared to 2D (Figure 6C). Viability was further lowered after 12 days of treatment, although still high in 3D (6 ± 5% for 2D vs 30 ± 12% and 12 ± 1% for PDOs in GelMA and GelSH, respectively, at 1 µM treatment). Such trends are in agreement with several studies that recapitulated BCa PDOs in various 3D matrices using BCa cell lines, and is linked to reduced drug diffusion [8] and ECM proteins modulating antimigratory and apoptotic effects [62]. This was seen for T47D and MCF-7 cell lines in HA hydrogels [63], decellularized ECM models [63] and topography-enhanced GelMA gels, although no studies could be found to date with patient-derived cells.

#### 3.2.2. Response to Doxorubicin, EP31670 and Paclitaxel

**Figure 7:**
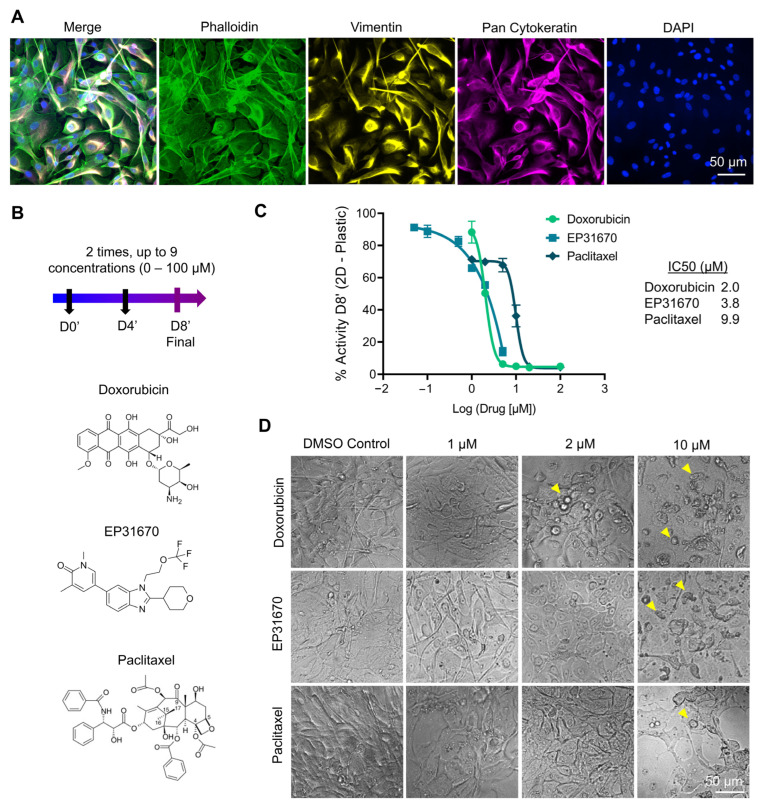
2D drug response from P#04F patient-derived cells. (**A**) Epifluorescence images from DAPI (blue), pan-cytokeratin (yellow), vimentin (magenta), phalloidin (green) staining epithelial origin of subcultured cells and epithelial-to-mesenchymal transition process. (**B**) Drug treatment schedule and drug structures. (**C**) IC_50_ doses responses (Means ± SE, *n* = 3). (**D**) Brightfield images of cell monolayers after 8 days of treatments. Yellow arrows show cell damage.

**Figure 8:**
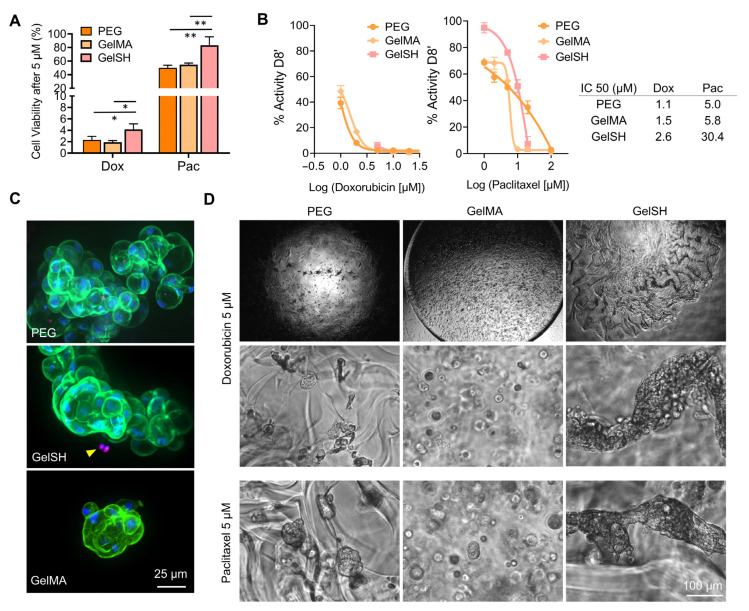
Doxorubicin and paclitaxel response on 3D PDO cultures in PEG, GelSH and GelMA biomaterials of similar stiffness (3 kPa) from P#04F patient-derived cells after 8 days of treatments (administered twice at day 0 and day 4). (* *p* < 0.01, ** *p* < 0.05). (**A**) Cell viability after 5 µM of doxorubicin (Dox) and paclitaxel (Pac). (**B**) IC_50_ doses responses. (**C**) Confocal images of immunofluorescence for DAPI (blue), γH2AX (magenta), Phalloidin (green) staining prior to drug testing showing undamaged organoids, yellow arrows show organoids damage. (**D**) Brightfield images of 3D PDO morphology in PEG, GelSH and GelMA gels 5 µM of doxorubicin (Dox) and paclitaxel (Pac).

A large variety of lines of treatments are available to BCa patients and are chosen according to pathological assessment and hormonal status. For triple negative BCa, neo-adjuvant cytotoxic therapy is offered, with doxorubicin/cyclophosphamide in combination followed by paclitaxel ± carboplatin. If residual cancer is present in the resected specimen, adjuvant capecitabine may be employed. Patients with HER2+ tumors are offered Trastuzumab in addition to the above chemotherapy. For HR+/HER2, tamoxifen or an aromatase inhibitor ± goserelin are the preferred options. Adjuvant chemotherapy (doxorubicin (adriamycin)/cyclophosphamide followed by paclitaxel) may be given if the patient is higher risk [64]. We thus sought to do a comparison of doxorubicin with paclitaxel, as recurrent agents in the standard of care of BCa. We also tested a non-traditional agent, EP31670 (also known as NEO2734), a pan BET/CBP/EP300 inhibitor, which exhibits a range of antiproliferative activity in several cell lines, with the highest activity observed in hematologic and prostate cancers [37]. Figure 7 shows treatment response of the three drugs (Figure 7B) on near-confluent monolayers of P#04F cells, which were cytokeratin- and vimentin-positive (Figure 7A), confirming epithelial origin and reflective of epithelial-to-mesenchymal transition seen in BCa cells. The patient cells responded to all three drugs (Figure 7C,D), with IC_50_ ranging from 3, 3.8 and 9.9 µM for doxorubicin, EP31670 and paclitaxel, respectively. Paclitaxel, compared to doxorubicin, was 5-fold less effective, which is line with previous studies and with what is seen in the clinic. For example, in the clinic even when receiving higher doses of paclitaxel (200 mg/m^2^ versus 75 mg/m^2^ for doxorubicin every 3 weeks), doxorubicin achieved better disease and symptom control than paclitaxel in advanced BCa first-line treatment [65]. It is worth noting that the efficacy difference between doxorubicin and paclitaxel was 5-fold in 2D, but it was 20–28-fold in any of the 3D hydrogels biomaterials (Figure 8A), emphasizing the significant differences in outcomes that may occur using 2D or 3D screening platforms [1]. Additional assessment in 3D showed that the BCa PDOs cultured in PEG and GelMA had similar IC_50_ for both drugs, yet BCa PDOs in GelSH had 1.7 and 5.2-fold increase in the IC_50_ compared to the UV-crosslinked GelMA, for doxorubicin and paclitaxel, respectively. This is likely due to differences in organoid size at treatment start, which is a limitation that needs addressing in future work, with GelSH presenting with complex structures over 500 µm in size. This also raises a problem for the field, namely that IC_50_, a model tool for commercial drug research and development in 2D may be unsuitable for 3D culture systems. For large spheroids in particular, IC_50_ curve fitness values differ significantly and are complicated by complex morphologies [66]. Conversely, Berrouet et al. showed how diffusivity, drug action mechanism and cell proliferation capabilities result in variable IC_50_ curves of 3D models [67]. In the future, other evaluation indexes and experimental tools could be employed, such as maximum inhibition and spheroid volume assessment, respectively. In addition, reduced drug potency in GelSH may also be the result of better ECM interaction with GelSH over the other two alternatives, as ECM drives a significant share of drug resistance in BCa and thus this needs to be investigated further.

Both drugs investigated here generated increased organoid disintegration and cell death with increasing concentrations (Figure 8D and Figure S6) although different morphologies were observed. This is in line with the different mechanisms of action of both drugs, where doxorubicin intercalates into DNA and disrupts DNA repair [68] while paclitaxel suppresses microtubule dynamics, blocking metaphase-anaphase transitions [69], making them ideal to be used in sequential combination, as is done in the clinic for ER+/PR+/HER2− and TNC subtypes [65,70]. The presence of ECM is a key contributor to therapy response [8,71,72,73] which needs urgent accounting in 3D models, especially in BCa where heterogeneity is one of the main resistance mechanisms of breast cancer therapy [74]. The incorporation of ECM-based matrices surrounding PDOs is likely to drastically change drug responses. In a study by Luo et al., for example, PDOs were made out of a case of adenomyoepithelioma of the breast reliant on self-assembled matrix-free spheroids [75]. While the diameter of the organoids successfully grew from 20 to 80 µm from 1 to 7 days of culture and decreased with increased drug concentrations, the effect of doxorubicin and paclitaxel were similar at similar doses (between 500 and 1000 nM) [75]. This is unlikely to recapitulate the physiological scenario, where ECM proteins would have reduced drug diffusion [8] and modulated apoptotic effects [62] and not line with clinical response for these drugs. In summary, our various ECM-containing hydrogels recapitulated a better BCa microenvironment, as shown by extended PDO growth, and were able to be used for drug response and potency assessment with standard-of-care therapies used in the clinic [65].

## 4. Conclusions

Three types of hydrogel-based biomaterials with biological and physicochemical characteristics similar to BCa native ECM were successfully used to encapsulate patient-derived cells from tumor tissue from patients with three highly common BCa subtypes. The functionalization of a synthetic PEG hydrogel with cell adhesion peptides, including RGD, GFOGER and DYIGSR, was key to support ex vivo organoid growth. The use of semi-synthetic matrices based on gelatin proved to be equally highly suited or even superior, with complex and large organoid structures and long-term growth at least to 2–3 weeks. While this is positive, more investigation needs to be done to ensure mixing prior to gelation and similarly-shaped organoids for reproducible drug testing purposes. The hydrogels could be tuned to provide stiffnesses relevant to most BCa tissues at early disease stage (1–6 kPa) while maintaining high viability. Yet, the traditional UV-crosslinked GelMA led to less growth and more rounded phenotypes than a novel thiolated-gelatin crosslinked by Michael-type click-chemistry to form GelSH hydrogels. The cells from all patients responded to model standard-of-care treatments, including doxorubicin and paclitaxel, with >2-fold drug resistance when cultured in all 3D configurations, compared to 2D monolayers. Paclitaxel was overall less effective than doxorubicin, a difference in response that can be seen in the clinic. Overall, the GelSH material provided the best organoid growth and structures and long-term viability of the BCa organoid structure, with the least drug potency. In future, when available, additional comparison studies with the original tumor will be helpful to delineate what engineered microenvironment best replicates the clinical scenario. By mimicking the ECM and stiffness of BCa and showing a differential drug response, we have shown how the hydrogel-based materials are valid, tunable platforms for successful ex vivo culture of patient tumor tissue and drug testing. As intratumor heterogeneity could be one of the main resistance mechanisms of breast cancer therapy, these versatile 3D hydrogel culture systems suited to patient-specific tissue culture hold great hope for the future of precision medicine.

## Figures and Tables

**Table 1:** BCa patient sample summary (all females).

ID	Type	Age	ER	PR	HER2−
P#01F	Fresh tumor from patient	32	−	−	+
P#02X	Xenograft	45	−	−	−
P#03F	Fresh tumor from patient	76	+	+	−
P#04F	Fresh tumor from patient	54	+	+	−

**Table 2:** 3D hydrogel systems assessed for BCa PDO growth.

Hydrogel	Manufacturing	Crosslinking	Standard Working Volume (µL) *
Polyethylene glycol (PEG) functionalized with DYIGSR, GFOGER and RGD	Bioprinting	Michael-Type	0.8–2.4
Gelatin methacryloyl (GelMA)	Manual	UV (365 nm)	45–65
Thiolated gelatin/4-armed PEG-maleimide (GelSH)	Manual	Michael-Type	20–60

* Note that a wider range of volumes may be obtained. These numbers only represent volumes that are typically used.

## Data Availability

All data needed to evaluate the conclusions in the paper are present in the paper.

## Supplementary Materials

The following supporting information can be downloaded at: https://www.mdpi.com/article/10.3390/pharmaceutics15010261/s1, Figure S1. Additional brightfield images of P#03F breast cancer patient-derived organoids grown in 3 kPa GelMA and GelSH gels. Yellow arrows show organoids., Figure S2. Additional brightfield images of BCa PDOs grown in 3 kPa and 6 kPa GelSH gels (P#04F), Figure S3. Brightfield and epifluorescence stitched images of entire GelSH hydrogels containing breast cancer patient-derived organoids after 12 days of culture after FDA/Hoechst/PI staining, showing overall morphology and live cells (green), cell nuclei (blue), dead cells (red) (P#04F), Figure S4. Doxorubicin response on breast cancer patient-derived cells grown in 2D (plastic) and 3D (matrix-free, Agar 1%), P#03F. (A) Doxorubicin treatment schedule. (B) IC50 dose responses for 2D cultures after 11 days in culture, followed by either 4, 8 or 12 days of doxorubicin treatments (D4’, D8’, D12’, respectively, means ± SEM, *n* = 3–5 technical replicates). (C) Brightfield images of 2D and 3D cultures after 11 days in culture and prior to drug treatments. (D) Cell viability after 12 days treatment with doxorubicin and resulting IC50 dose responses (Means ± SE, *n* = 3–4). (E) Brightfield images after 4 days of doxorubicin treatment, Figure S5. Additional brightfield images of 3D PDO morphologies in GelSH and GelMA gels of 3 kPa after 8 days of treatment (P#03F), Figure S6. Additional brightfield images of 3D PDO morphologies in GelSH gels (P#04F) after 8 days of treatment of doxorubicin and paclitaxel.
