# Supplementary material for: GelMA, Click-Chemistry Gelatin and Bioprinted Polyethylene Glycol-Based Hydrogels as 3D Ex Vivo Drug Testing Platforms for Patient-Derived Breast Cancer Organoids
Source: Pharmaceutics. 2023 Jan 12;15(1):261. doi: 10.3390/pharmaceutics15010261 (PMC9867511; doi:10.3390/pharmaceutics15010261)
Supplement: Supplementary file 1 [file pharmaceutics-15-00261-s001.zip › pharmaceutics-2102625-supplementary.pdf]

pharmaceutics

Supplementary Materials: GelMA, Click-Chemistry Gelatin and Bioprinted Polyethylene Glycol-Based Hydrogels as 3D Ex Vivo Drug Testing Platforms for Patient-Derived Breast Cancer Organoids

Nathalie Bock, Farzaneh Forouz, Luke Hipwood Julien Clegg Penny Jeffery, Madeline Gough, Tirsa van Wyngaard, Christopher Pyke, Mark N. Adams, Laura J. Bray, Laura Croft, Erik W. Thompson, Thomas Kryza and Christoph Meinert

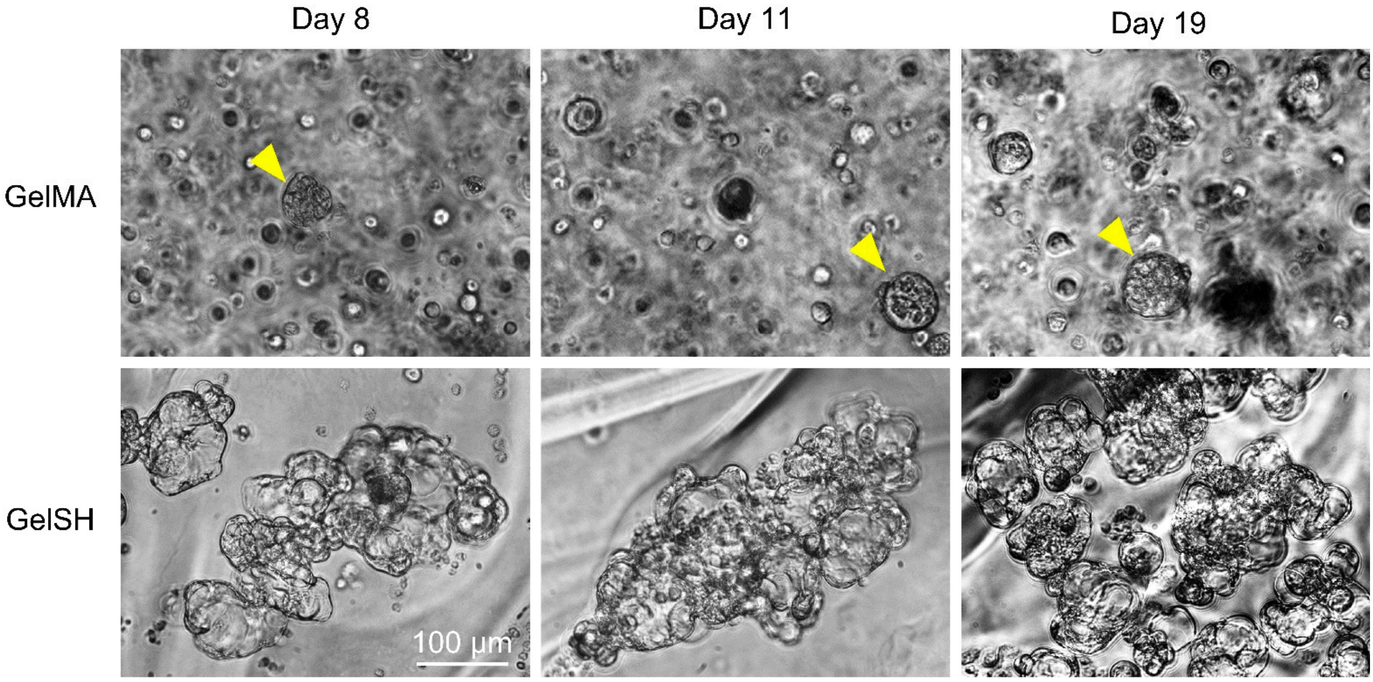

Figure S1. Additional brightfield images of P#03F breast cancer patient-derived organoids grown in 3 kPa GelMA and GelSH gels. Yellow arrows show organoids.

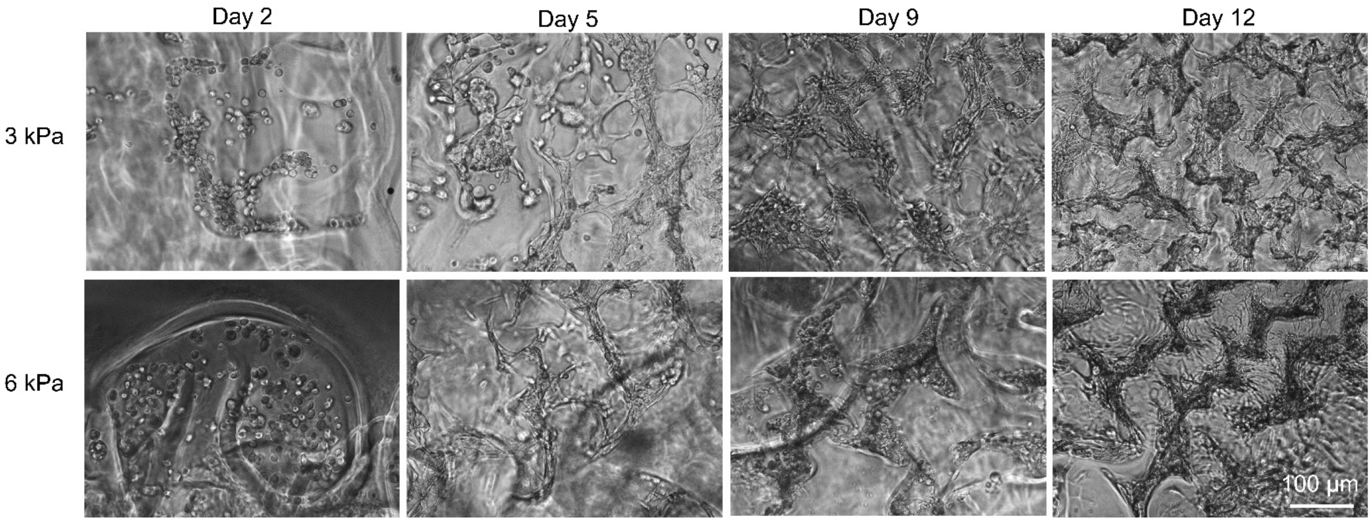

Figure S2. Additional brightfield images of BCa PDOs grown in 3 kPa and 6 kPa GelSH gels (P#04F).

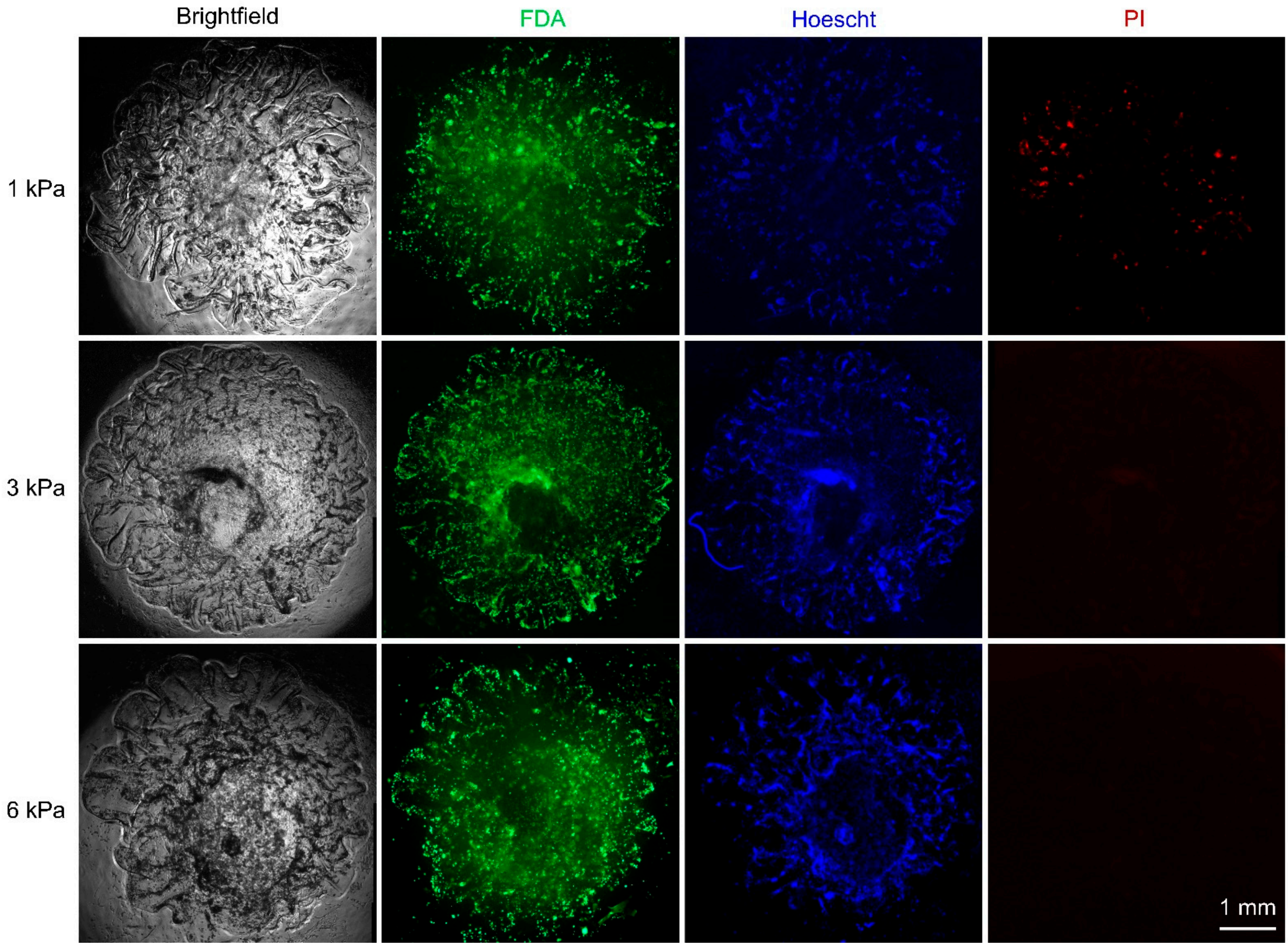

Figure S3. Brightfield and epifluorescence stitched images of entire GelSH hydrogels containing breast cancer patient-derived organoids after 12 days of culture after FDA/Hoechst/PI staining, showing overall morphology and live cells (green), cell nuclei (blue), dead cells (red) (P#04F).

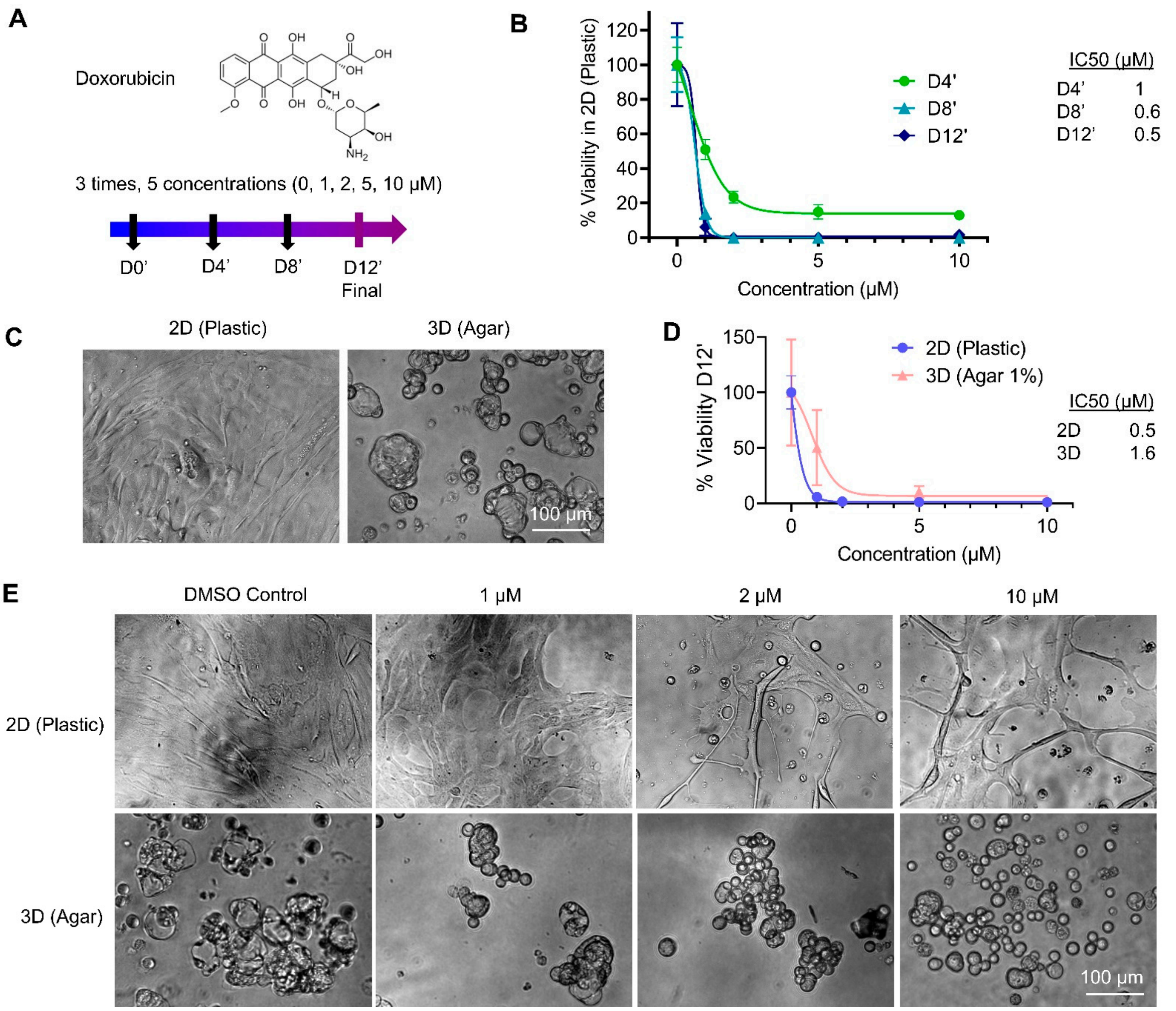

Figure S4. Doxorubicin response on breast cancer patient-derived cells grown in 2D (plastic) and 3D (matrix-free, Agar 1%), P#03F. (A) Doxorubicin treatment schedule. (B) IC50 dose responses for 2D cultures after 11 days in culture, followed by either 4, 8 or 12 days of doxorubicin treatments (D4′, D8′, D12′, respectively, means ± SEM, n = 3–5 technical replicates). (C) Brightfield images of 2D and 3D cultures after 11 days in culture and prior to drug treatments. (D) Cell viability after 12 days treatment with doxorubicin and resulting IC50 dose responses (Means ± SE, n = 3–4). (E) Brightfield images after 4 days of doxorubicin treatment.

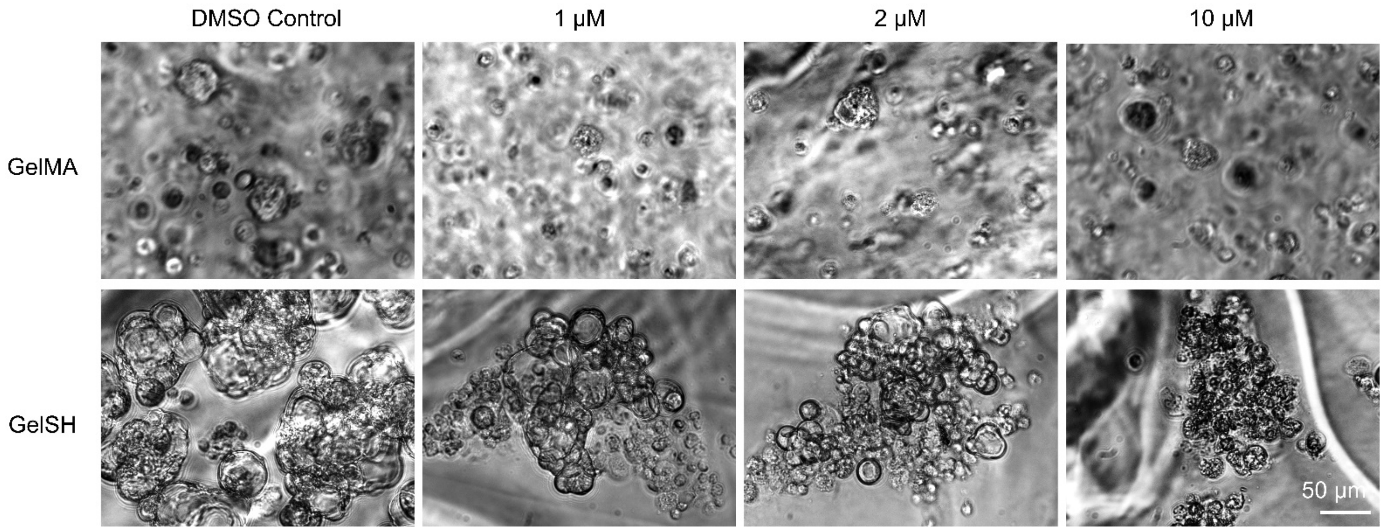

Figure S5. Additional brightfield images of 3D PDO morphologies in GelSH and GelMA gels of 3 kPa after 8 days of treatment (P#03F).

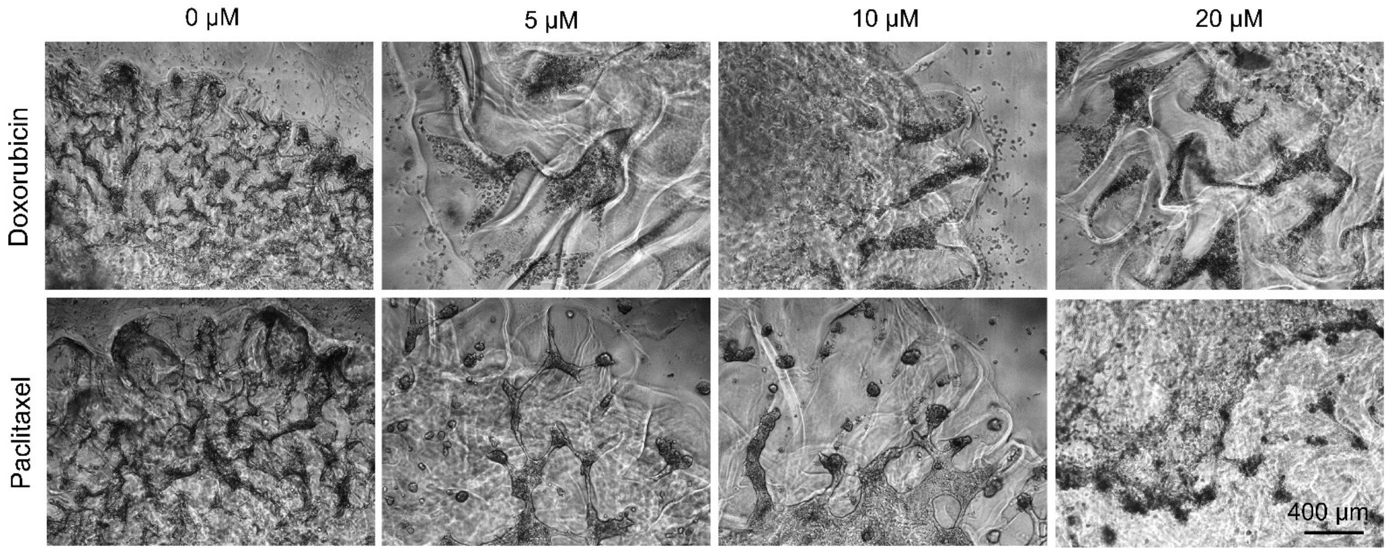

Figure S6. Additional brightfield images of 3D PDO morphologies in GelSH gels (P#04) after 8 days of treatment of doxorubicin and paclitaxel
